# Human Infant Fecal Microbiota Differentially Influences the Mucosal Immune Pathways Upon Influenza Infection in a Humanized Gnotobiotic Pig Model

**DOI:** 10.1007/s00284-024-03785-8

**Published:** 2024-07-14

**Authors:** Jennifer Schrock, Ming Yan, Sara Dolatyabi, Veerupaxagouda Patil, Ganesh Yadagiri, Sankar Renu, Anikethana Ramesh, Ronna Wood, Juliette Hanson, Zhongtang Yu, Gourapura J. Renukaradhya

**Affiliations:** 1 Department of Animal Sciences, Center for Food Animal Health (CFAH), College of Food Agricultural and Environmental Sciences (CFAES), 1680 Madison Avenue, Wooster, OH 44691 USA; 2 Department of Animal Sciences, CFAES, The Ohio State University, Columbus, USA; 3 Center of Microbiome Science, The Ohio State University, Columbus, OH USA

## Abstract

In this study, we evaluated the impact of human gut microbiota on the immune pathways in the respiratory tract using a gnotobiotic (Gn) piglet model. We humanized piglets with rural and urban infant fecal microbiota (RIFM and UIFM, respectively) and then infected them with a H1N1 swine influenza virus. We analyzed the microbial diversity and structure of the intestinal and respiratory tracts of the piglets before and after the influenza virus infection and measured the viral load and immune responses. We found that the viral load in the upper respiratory tract of UIFM transplanted piglets was higher than their rural cohorts (RIFM), while virus-specific antibody responses were comparable. The relative cytokine gene expression in the tracheobronchial (respiratory tract) and mesenteric (gastrointestinal) lymph nodes, lungs, blood, and spleen of RIFM and UIFM piglets revealed a trend in reciprocal regulation of proinflammatory, innate, and adaptive immune-associated cytokines as well as the frequency of T-helper/memory cells, cytotoxic T cells, and myeloid immune cell subsets. We also observed different phylum-level shifts of the fecal microbiota in response to influenza virus infection between the two piglet groups, suggesting the potential impact of the gut microbiota on the immune responses to influenza virus infection and lung microbiota. In conclusion, Gn piglets humanized with diverse infant fecal microbiota had differential immune regulation, with UIFM favoring the activation of proinflammatory immune mediators following an influenza virus infection compared to their rural RIFM cohorts. Furthermore, Gn piglets can be a useful model in investigating the impact of diverse human microbiota of the gastrointestinal tract, probably also the respiratory tract, on respiratory health and testing specific probiotic- or prebiotic-based therapeutics.

## Introduction

Annual epidemics of influenza result in 3 to 5 million cases of severe illness and 290,000 to 650,000 deaths globally [1]. Strikingly, about 99% of the deaths occur in children under the age of two years with serious health complications due to influenza [2]. Dysbiosis of the microbiota of the upper respiratory tract was reported in children with influenza A virus infection [3]. The gut microbiota plays an important role in maintaining immune homeostasis and good health and can influence the outcome of infections [4]. The types of microorganisms colonizing in infants during the first year of life can profoundly impact the outcome of immune responses for the rest of their life [5]. The diverse commensal microbes influence the immune development and function of the mucosal immune system and inhibit the colonization of pathogenic organisms [6]. Influenza is a disease of humans and some species of mammals and birds globally [7]. Humans and piglets are the natural hosts of influenza causing similar acute respiratory diseases mediated by secreting inflammatory mediators, causing fever, body aches, and fatigue [8, 9]. Recent research has been focusing on gut microbiota transplantation therapy to mitigate the severity of influenza, due to the low to moderate efficacy of seasonal influenza vaccines against disease and virus transmission [10]. Studies have revealed that a diverse and healthy commensal gut microbiome activates the innate immune pathways mediated via Toll-like receptors [11] and protects the host against influenza virus infection [12]. The nose/throat microbiota diversity before influenza virus infection is associated with influenza symptoms and duration of virus shedding in the host [13].

The diversity of intestinal microbiota is attributed mainly to a host of factors including the environment where people live, and gut microbes constantly interact with host immune cells. Gut microbes and their metabolites influence not only the health of the gut but also the respiratory tract [14–16]. Rural children have a lower prevalence of asthma and allergic sensitization by four- to six-fold than their urban cohorts, attributed to early life exposure to diverse allergens, microbes, and endotoxins, which have a profound influence on building the healthy immune system [17]. Studies using a mouse model provide crucial insights into the mechanism(s) regulating the immune systems mediated by the gut microbiota [18, 19]. However, the applicability of the microbiota-induced immune response in mice to humans for translational studies is questionable [20]. Thus, to understand the role of gut bacterial species and their diversity on respiratory tract immune profiles, a suitable large animal model that closely mimics the growth and colonization of most human gut bacteria is essential [21].

Swine are considered an appropriate animal model for influenza research over rodents because, unlike mice, pigs are a natural host for influenza as humans, and their anatomical, immunological, physiological, and genetic compositions are comparable to humans [22–24]. Comparison between gnotobiotic pig and mouse for immune and microbiome research is provided in a table (Supplementary Table S1). We demonstrated in a previous study that gnotobiotic (Gn) piglets humanized with rural and urban infant human fecal microbiota (RIFM and UIFM) harbored microbial diversity and composition comparable to the original IFM inoculum and differentially modulated the mucosal immune development [21]. In the present study, we humanized Gn piglets with RIFM and UIFM collected in that study [21] and further examined how the two types of diverse fecal microbiota modulate immune responses to influenza virus infection, and whether immune pathways correlate to specific groups of microbes in the host.

## Materials and Methods

### Fecal Microbiota Sample Collection

The details of collection of fecal samples from healthy infants from Amish (rural) and non-Amish (urban) families in accordance with the sampling protocol approved by The Ohio State University Institutional Review Board (IRB#2015H3281) were provided in our previous study [21]. We used five each of apparently healthy infants from rural and urban families. The Amish families had farm animals (cattle, sheep and/or horses) and pets (dog and/or cat), while the non-Amish households were from families located in the Wooster city limits with no contact with livestock but had pets. For this study, infants who were born through natural vaginal delivery were enrolled. Fecal samples were collected from fresh soiled diapers as described previously [21, 25, 26]. Briefly, the parents placed the fecal material in a tube containing glass bead for homogenization, then filled the tube with sterile anaerobic media. Tubes were kept on ice and immediately transported to the lab. Inside of an anaerobic chamber, the sample was homogenized vigorously, 15% sterilized glycerol was added, and 1 mL aliquots were prepared and stored at −80 °C.

### Fecal Microbiota Humanization and Influenza Virus Infection of Gn Piglets and Sample Collection

A healthy pregnant sow (gestation day 105) procured from The Ohio State University swine herd was kept in our swine isolation facility for one week rest period. Gn piglets were delivered by cesarean section as described previously [27]. Piglets were kept and cared in individual temperature-controlled sterile Gn isolators and fed with sterile infant milk formula as described [21]. The male and female piglets were randomly allocated into two groups (n = 4 or 5 per group). Rural and urban fecal microbial inocula were prepared freshly by pooling five each of Amish (rural) infant fecal microbiota (RIFM) aliquots (1.0 mL each) and the other being a mixture of the five non-Amish (urban) infant fecal microbiota (UIFM) aliquots. The pooling of the microbiota took place inside an anaerobic chamber to maintain anaerobiosis. Each fecal inoculum was mixed with a 50 ml sterile infant milk formula and delivered orally once to individual piglets of each group at 8 days of age. An exactly similar procedure and same pooled five infants RIFM and UIFM were used in an earlier pig transplantation study for microbial and immune analysis [21]. Due to limited space to handle many pigs in our germ-free isolators, we did not use those groups again as controls in this study.

**Fig. 1:**
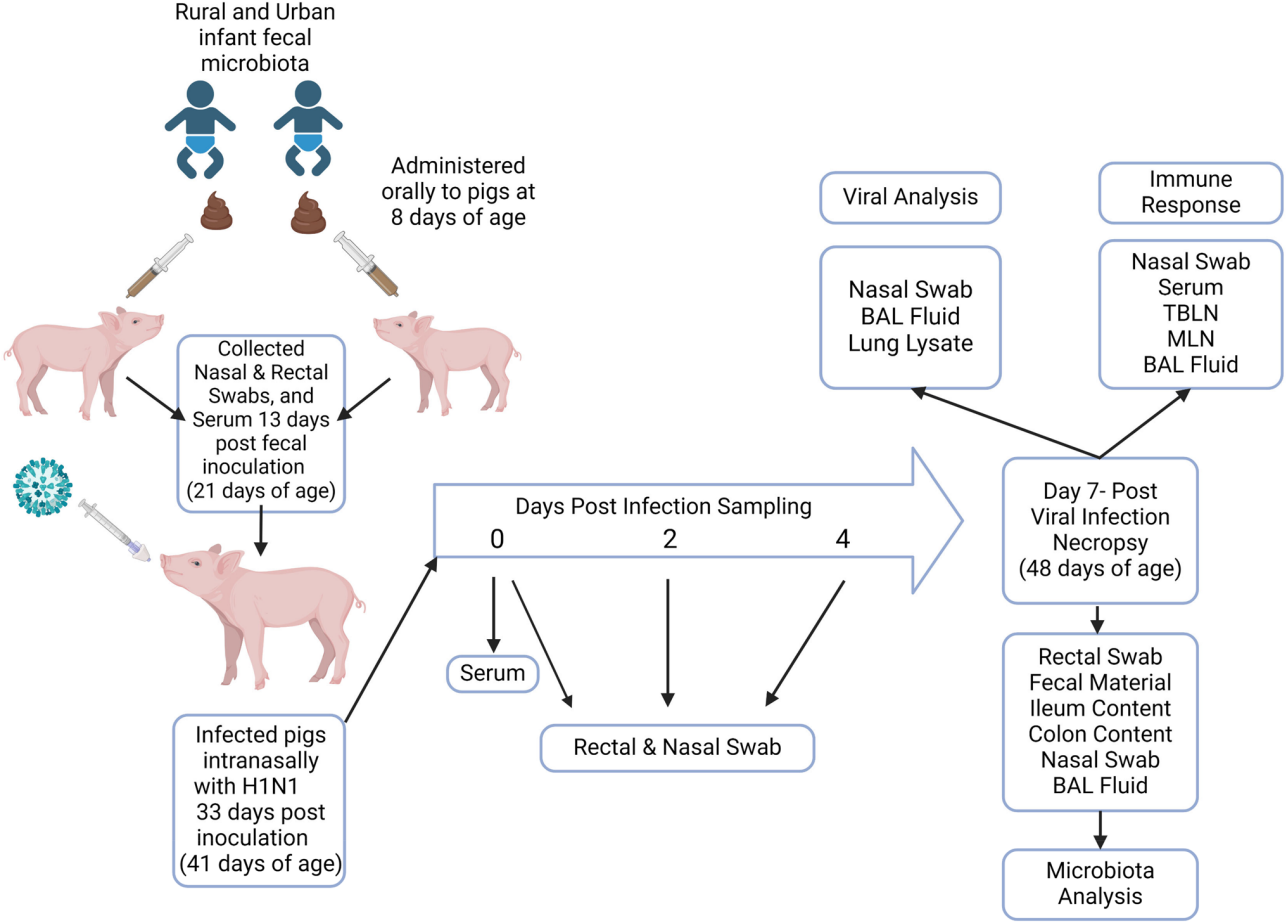
Schematic illustration of the experimental design, sampling time points, and information about the immune and microbial analysis. Gn piglets were humanized with fecal microbiota of rural and urban human infants and infected with a zoonotic swine influenza H1N1 virus

Gut microbiota-humanized piglets were infected with a swine influenza virus H1N1 (A/Swine/OH/24366/2007) [21, 28] by inoculation of 2 × 10^7^ TCID_50_ (tissue culture infectious dose) virus in 1 mL as intranasal drops at 41 days of age (33 days post-fecal inoculation). Piglets were monitored twice daily for clinical influenza signs (fever, lethargy, anorexia, and labored breathing). Nasal and fecal swab samples were collected at days post-viral infection (DPI) 0, 2, 4 and 7 for microbiota analysis and to determine the viral load. On the day of necropsy (DPI7), piglets were given a sedative, TKX (telazol, xylazine, and ketamine), and blood was collected for isolating peripheral blood mononuclear cells (PBMCs) and euthanized by injecting sodium pentobarbital by intracardiac route into the heart. We collected bronchoalveolar lavage (BAL) fluid, tracheobronchial lymph nodes (TBLN), and mesenteric lymph nodes (MLN) for isolating mononuclear cells (MNCs) as described earlier [29]. Spleen, lung, TBLN, and MLN tissue samples were collected in *RNAlater* for the analysis of cytokine gene expression. Nasal wash, BAL fluid, ileum, and colon contents were used for microbiota analysis **(**Fig. 1**)**. In this study, we did not include some of the control groups such as a group treated with IFM but no influenza infection and a group that was not infected or treated with IFM, because using the same infant fecal material we performed the detailed analysis including those groups for colonized microbiota species in a previous study in 2019 [21].

### Determining the Influenza Virus Load in the Respiratory Tract

Estimation of the replicating virus load was performed by cell culture method as described previously [29]. Briefly, tenfold diluted nasal swab fluid and BAL fluid samples were subjected to virus titration using madin-darby canine kidney epithelial (MDCK) cells incubated for 48 h at 37 °C in 5% CO_2_ incubator. Cells were immunostained with influenza virus nucleoprotein-specific primary antibody (CalBioreagents, CA) followed by treatment with the secondary antibody AlexaFluor 488-conjugated goat anti-mouse IgG (H + L) (Life technologies, CA). The green fluorescence signals were read under a fluorescent microscope and virus titers were calculated in 50% TCID_50_/ml as described previously [29].

### Influenza Virus-Specific Antibody Analysis

The swine influenza virus H1N1-specific IgG and IgA antibodies in serum, BAL fluid, and nasal swab samples were determined by ELISA as described previously [30]. Briefly, 96-well plates were coated with pretitrated killed virus antigens overnight (10 μg/mL) and blocked with 5% skim milk powder containing 0.05% Tween-20. Plates were washed and then diluted test samples in PBS containing 2.5% skim milk powder and Tween-20 were added to marked duplicate wells, incubated for 2 h at room temperature (RT), washed, and secondary antibody horse radish peroxidase-conjugated goat anti-pig IgG (KPL, MD, USA) or goat anti-pig IgA (Bethyl laboratories Inc., TX, USA) was added. The antigen and antibody interactions were detected by using 1:1 mixture of peroxidase substrate solution B and TMB peroxidase substrate (KPL, MD, USA). The reaction was stopped by adding 1.0 M phosphoric acid after 10 min of incubation, and optical density was measured at 450 nm using a Spectramax microplate reader (Molecular devices, CA, USA).

### Flow Cytometry Analysis

PBMCs, BAL cells, and TBLN and MLN MNCs were immunostained to determine the frequency of cytotoxic T lymphocytes (CTLs) (CD3^+^CD4^−^CD8α^+^β^+^), T-helper/memory cells (CD3^+^CD4^+^CD8α^+^β^−^), and myeloid cells (CD3^−^ non-T cells and CD3^−^CD172a^+^ cells) using specific immune markers and analyzed as described earlier [21, 29, 31]. The cells were fixed and 100,000 events were acquired (BD Aria II flow cytometer, BD Biosciences, CA) and the data were analyzed using FlowJo software (Tree Star, Palo Alto, CA). The specific anti-pig and their respective isotype control monoclonal antibodies used for immunostaining lymphocytes and myeloid cells were either purified, biotin or fluorochrome labeled, that include CD3 (Southern biotech, AL), CD4α, CD8α, monocyte/granulocyte CD172a (Southern biotech, AL), and CD8β chain (BD Pharmingen, CA) followed by the addition of anti-isotype-specific secondary antibody or streptavidin fluorochrome.

### Quantitative Reverse Transcription PCR (RT-qPCR) Analysis for Immune Gene Expression

Total RNA was extracted from samples using the Trizol reagent (Invitrogen, Carlsbad, CA, USA) by following the manufacturer's protocol. The integrity and purity of the extracted RNA were assessed via spectrophotometry (NanoDrop 2000, Thermo Fisher Scientific, Waltham, MA, USA), ensuring the A260/A280 ratio was between 1.8 and 2.1. To remove any potential DNA contamination, RNA samples were treated with DNase I (Sigma-Aldrich, St. Louis, MO, USA).

Quantitative real-time PCR was conducted using the SYBR Green PCR Master Mix (Applied Biosystems, Foster City, CA, USA) on a One-Step Real-Time PCR System (Applied Biosystems, Foster City, CA, USA). Each 20 µL reaction mixture included 10 µL of SYBR Green Master Mix, 1 µL of each primer (10 µM), 2 µL of cDNA, and 6 µL of nuclease-free water. The sequence details of primers used are provided (Supplementary Table S2). The thermal cycling conditions were as follows: initial denaturation at 95 °C for 10 min, followed by 40 cycles of denaturation at 95 °C for 15 s, and annealing/extension at 60 °C for 1 min. Relative expression levels of the target genes were calculated using the 2 − ΔΔCT method, with β-actin serving as the reference gene as described previously [30, 32–34].

### Metataxonomic Analyses of Microbiota

Metagenomic DNA extraction, amplicon library preparation (the V3-V4 regions of the 16S rRNA gene), paired-end sequencing (2 × 300 bp), and subsequent sequence data processing and analyses were the same as described in a previous study [21]. Briefly, with QIIME2 (version 2021.4) [35], the amplicon sequencing reads were demultiplexed, trimmed off the adapter and primers, quality filtered (Q-score ≥ 25), denoised, and merged using the DADA2 plugin [36]. After removing chimeric sequences, DADA2 clustered the sequences into amplicon sequence variants (ASVs) and generated the ASV feature table. The naive Bayes classifier was trained against the targeted region (V3-V4) of the SILVA database (release v138) [37] and then used to taxonomically assign the ASVs. The alpha- and beta-diversity were analyzed using the Phyloseq package [38] in R [39], with the number of ASVs rarefied to 4,000 per sample for alpha-diversity analysis.

### Statistical Analysis

For the immune response data, statistical comparisons were made between RIFM and UIFM humanized piglet groups (i.e., RIFMP and UIFMP) for each designated time point, sample type, and type of experiment throughout the study unless specified otherwise by using JMP Pro 15 (SAS; Cary, NC), GraphPad Prism 9.5 (San Diego, CA), and RStudio (Boston, CA). The virus titer, antibody levels, cytokine mRNA expression, and flow cytometry data were analyzed by using non-parametric Mann–Whitney test. The data were presented as the mean ± SEM of each piglet group. Significance was declared at *P* < 0.05. Asterisks denote significant difference (*P < 0.05, **P < 0.01, and ***P < 0.001).

For the microbiota data, Shannon diversity index values were compared between RIFMP and UIFMP at each time point with the Wilcoxon rank sum test. The two piglet groups were compared for their overall microbiota at each time point with principal coordinates analysis (PCoA) based on Bray–Curtis dissimilarities, and significance of difference was tested with permutational multivariate analysis of variance (PERMONAVA) in R using the adonis function of the vegan package [40] in R with 999 permutations. The ASVs with a relative abundance of less than 0.01% and detected in less than 10% of the piglets were filtered out and then the major ASVs were collapsed into genera. Differentially abundant genera between the two piglet groups and before and after the influenza virus infection were analyzed with ANCOM-BC with the following settings: zero_cut = 0.70, lib_cut = 0, struc_zero = TRUE, neg_lb = TRUE, tol = 1e-5, max_iter = 100, conserve = TRUE, and alpha = 0.05. The *P*-values were corrected for multiple comparison with the Holm–Bonferroni method, and an adjusted *P*-value < 0.05 was considered significant. Kendall’s rank correlation was used to determine the association of the relative abundance of bacterial genera with immune cell counts and the expression of cytokines. The correlation results were visualized with the ComplexHeatmap package in R [41].

## Results

### The Diverse Infant Microbiota Influences the Influenza Virus Load in the Upper Respiratory Tract But Not the Antibody Responses in IFM Humanized Piglets.

**Fig. 2:**
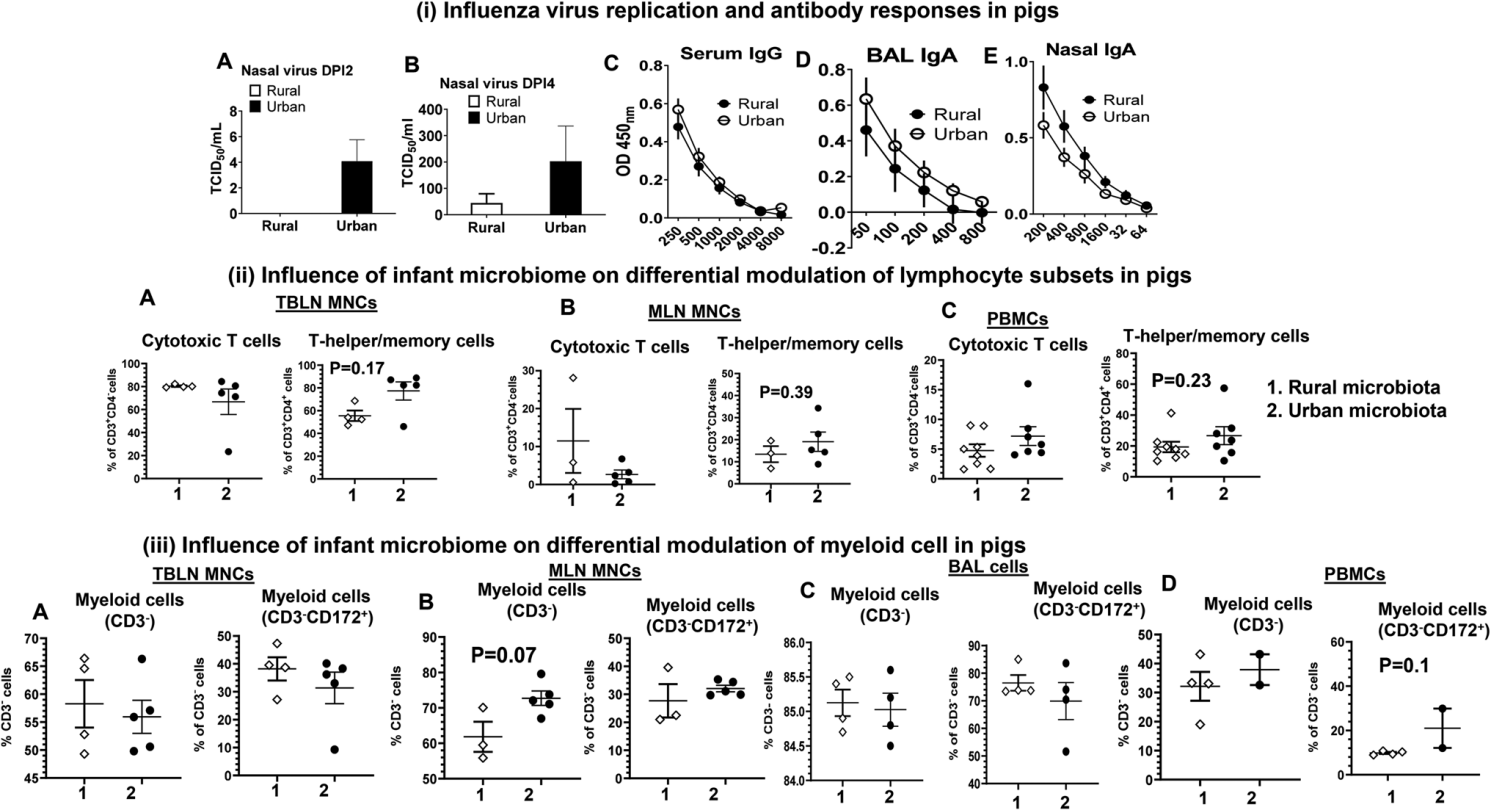
Impact of diverse rural and urban infant gut microbiota on influenza virus replication, antibody, and cellular immune responses in piglets. (i) Influenza virus replication and antibody responses in pigs: Nasal swab samples collected at DPI 2 (**a**) and DPI 4 (**b**) were analyzed for the virus load in nostrils. Virus-specific antibody responses at DPI 7: Serum IgG (**c**), BAL IgA (**d**), and Nasal IgA (**e**) were analyzed by ELISA. (ii) Influence of infant microbiome on differential modulation of lymphocyte subsets: Piglets were euthanized at DPI 7 and the isolated TBLN MNCs (**a**), MLN MNCs (**b**), and PBMCs (**c**) were immunolabeled and analyzed by flow cytometry to determine the frequencies of cytotoxic T cells (CD3^+^CD4^−^CD8α^+^CD8β^+^) and T-helper/memory cells (CD3^+^CD4^+^CD8α^+^CD8β^−^). (iii) Influence of infant microbiome on differential modulation of myeloid cells: Piglets were euthanized at DPI 7 and the isolated TBLN MNCs (**a**), MLN MNCs (**b**), BAL cells (**c**), and PBMCs (**d**) were immunolabeled and analyzed by flow cytometry to determine the frequencies of non-T cells (CD3^−^ Myeloid cells) and CD172^+^ Myeloid cells. Data represent the mean value ± SEM (4—5 piglets). Statistical analysis was carried out using Mann–Whitney test for comparison in all the graphs and only substantial changes between the groups were marked with the P-value.

The RIFM and UIFM humanized Gn piglets (RIFMP and UIFMP) were allowed to colonize with the IFM microbes for 33 days before being infected with a zoonotic swine influenza virus. Some of the UIFMP had mild fever (above 103°F) and none of their RIFMP cohorts had any fever. However, we did not observe any visible influenza-associated clinical signs in any of the piglets. This is consistent with earlier studies in which pigs aged 10–12 weeks infected with the same H1N1 viral strain only by intranasal route did not cause apparent disease, while infection by both intranasal and intratracheal routes  induced influenza signs and fever for 3–4 days including lung inflammation [29, 42]. The replicating infectious virus titers were measured in the nasal swab samples at various DPI and in BAL fluid at DPI 7. The virus was detectable at DPI 2 and 4, but not at DPI 7 in nasal swab and in BAL fluid. In UIFMP group, we detected higher viral titers at both DPI 2 and 4 than their RIFMP cohorts (Fig. 2i a, b and data not shown). However, influenza virus-specific systemic IgG and BAL and nasal IgA antibody responses were comparable in both the RIFMP and the UIFMP groups (Fig. 2i c, d, e).

### Both the Diverse Microbiota and Influenza Virus Infection Modulate Lymphoid and Myeloid Immune Cell Populations in Piglets.

The immune cells isolated from piglets were immunostained using specific anti-pig cell surface markers to identify the frequencies of lymphoid and myeloid cell subsets. We followed the standard cell gating strategy to delineate the cytotoxic T lymphocytes (CTLs), T-helper/memory cells and two myeloid cell subsets, and a schematic showing how the immune cells were gated for different phenotypic markers is presented [Supplementary Fig. S1). The two piglet groups had differential activation of immune cells in terms of relative abundance of CTLs and T-helper/memory cells in TBLN, MLN, and PBMCs [Fig. 2ii]. Particularly, T-helper/memory cells frequency tended to increase in UIFMP compared to their cohort RIFMP (Fig. 2ii a, b, c). The frequency of non-T cells (CD3^−^) and myeloid cells (CD3^−^CD172a) in the mucosal lymph nodes (TBLN and MLN), lungs, and blood was delineated [Fig. 2iii]. We observed an increased trend in the frequency of non-T cells (myeloid cells CD3^−^) in MLN and CD172a^+^ myeloid cells in PBMCs and a numerical decrease in TBLN and BAL cells (Fig. 2iii a, b, c, and d).

### Cytokine Gene Expression is Differentially Modulated in the Mucosal Tissues of Diverse IFM-Colonized Piglets.

**Fig. 3:**
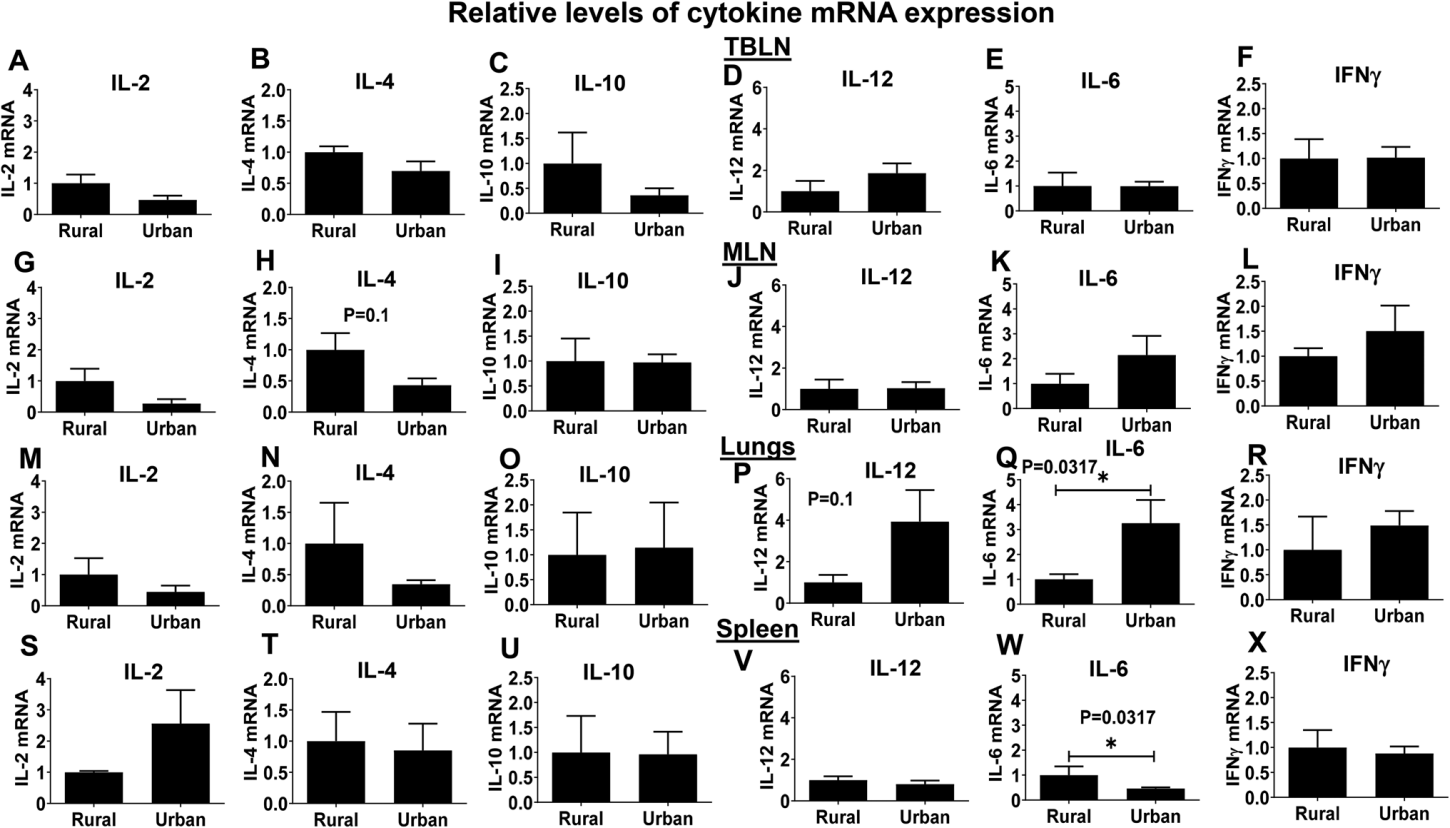
Relative gene expression of cytokines in the mucosal and systemic sites of piglets mediated by diverse gut microbiota and influenza virus infection. At DPI 7, animals were euthanized and RNA extracted from TBLN, MLN, lungs and spleen was subjected to analysis for the expression of mRNA encoding cytokines: (A, G, M, S) IL-2; (B, H, N, T) IL-4; (C, I, O, U) IL-10; (D, J, P, V) IL-12, (E, K, Q, W) IL-6; and (F, L, R, X) IFNγ by RT-qPCR. Data are presented as the mean ± SEM of each piglet group (3—5 piglets). Statistical analysis was carried out using Mann–Whitney test for comparison. Asterisks denote significant differences (*P < 0.05)

In TBLN of RIFMP compared to UIFMP group, we observed an increased trend in the expression of cytokines mRNA IL-2, IL-4, and IL-10, reduced trend in IL-12, and the comparable expression levels of IL-6 and IFNγ (Fig. 3a–f). In MLN of RIFMP compared to UIFMP group, we observed an increased trend in the expression of cytokines mRNA IL-2 and IL-4, reduced trend in IL-6 and IFNγ, and comparable levels of IL-10 and IL-12 expressions (Fig. 3g–l). In the lungs of RIFMP compared to UIFMP group, we observed an increased trend in the expression of mRNA of cytokines IL-2 and IL-4, reduced trend in IL-12 and IL-6 (significant P < 0.05), and comparable levels of IFNγ expression (Fig. 3m–r). In spleen of RIFMP compared to UIFMP group, we observed a reduced trend in the expression levels of cytokine mRNA IL-2, significantly (P < 0.05) increased IL-6, and comparable levels in the expression of IL-4, IL-10 IL-12 and IFNγ cytokines (Fig. 3s–x).

### Influenza Virus Infection Differently Affects the Fecal, Intestinal, and Respiratory Tract Microbiotas in RIFM vs. UIFM Piglets.

Shannon–Wiener diversity index of the fecal microbiota did not differ between the two piglet groups (RIFMP vs. UIFMP), or before and after the viral infection (Fig. S5), corroborating the observation of our previous study [21]. The overall fecal microbiota did not differ between RIFMP and UIFMP at day 22 but differed at day 40 (day of viral infection, prior to infection) and day 42 (2 days post-viral infection), and then converged at day 47 (7 days post-viral infection) (Fig. S2). This is consistent with our previous study in which significant difference in overall fecal microbiota was not observed until 5 weeks post-IFM humanization [21]. We acknowledge the small sample size that might have prevented robust microbiota comparison between the two groups of piglets.

Some individual bacterial taxa appeared to differ in relative abundance between the two piglet groups and responded differently to the viral infection. Bacteroidetes, Firmicutes, and Proteobacteria are the major phyla and Actinobacteria and Verrucomicrobia are the minor phyla of bacteria detected in the fecal samples over the course of the experiment (Fig. S3A). The two groups of piglets differed in relative abundance of the above bacterial phyla both before and after the influenza virus infection. At day 22, the rural group had a significantly higher relative abundance of Proteobacteria than in the urban group, while the opposite was true for Bacteroidetes, Firmicutes, and Verrucomicrobia. By day 40 (the same day of, but post, influenza virus infection), Firmicutes and Bacteroidetes gained abundance at the expense of Proteobacteria in the rural group, while in the urban group, only Firmicutes gained abundance at the expense of Bacteroidetes and Verrucomicrobia.

The response of fecal microbiota to influenza virus infection was evaluated at the genus level. The genera *Clostridium, Dorea, Klebsiella,* and *Streptococcus* and one unclassified genus of the family *Mogibacteriaceae* were found to be significantly more abundant in the urban than in the rural groups after the viral infection (Fig. S6).

The nasal microbiota also differed between the two piglet groups and underwent different shifts at the phylum level (Fig. S3B). Notably, on day 40 (day of viral infection, prior to infection), the rural group had more Bacteroidetes but less Actinobacteria and Proteobacteria than the urban group. Interestingly, by day 42 (2 days post-influenza virus infection), Firmicutes became the overwhelmingly dominant phylum in both piglet groups, with the rural group still having less Actinobacteria and Proteobacteria. Four days following viral infection, the rural group had little phylum-level shift, but the urban group had increased Proteobacteria and Actinobacteria but decreased Firmicutes. By the end of the experiment, 7 days post-influenza virus infection, the phylum-level profiles of the nasal microbiota in both groups mostly recovered, except for decreased Proteobacteria and detection of bacteria that could not be classified to any known phyla. Similar to the fecal microbiota, the nasal microbiota of the two piglet groups underwent different phylum-level shifts after influenza virus infection, which might reflect a difference in the nasal microbiota. It should be noted that the Gn piglets were humanized with infant fecal microbiota, but not nasal microbiota. Thus, their respiratory tract could only be colonized with bacteria from the fecal microbiota and the respiratory microbiota probably differed from that typically found in piglets.

At the end of the experiment, which was 7 days after the viral infection, when piglets were euthanized, colon, ileum, and bronchoalveolar lavage fluid were sampled for microbiota analysis. The rural group had more Proteobacteria but less Firmicutes in the lung than the urban group (Fig. S3C). In the colon, the rural group had more Bacteroidetes and Firmicutes but less Proteobacteria and Verrucomicrobia than the urban group. The two piglet groups had similar ileal phylum-level profiles. Without a control group that was not infected with influenza virus, we could not determine if the two piglet groups had different microbiota in their lung and intestines before influenza virus infection and how influenza virus infection might have caused different alterations of the lung and intestinal microbiota. However, the different phylum-level shifts of the fecal microbiota in response to influenza virus infection between the two piglet groups may also suggest different microbiota alterations in the lung and intestines. In addition, a longitudinal distribution of relative abundance of bacterial phyla detected in nasal passage at day 20 to 47, and day 47 in lungs, ilium, and colon is shown (Fig. S4).

### Some *Bacteria* in the Feces and Colon Content Correlate with Immune Cells and Immunoglobins.

**Fig. 4:**
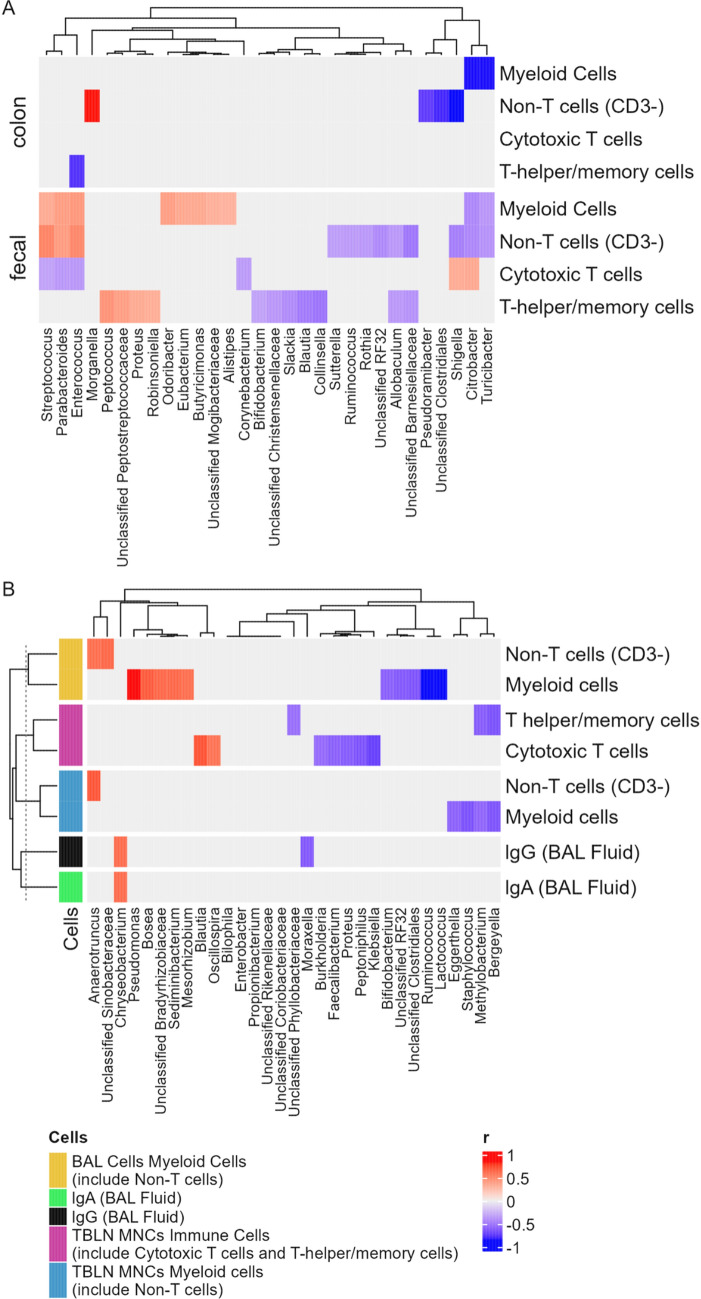
Kendall’s rank correlation between the relative abundance of bacterial genera of the piglet microbiota and select immunity metrics. **a** Correlation between the abundance of fecal bacterial genera (at the end of the experiment, DPI 7) and colonic bacterial genera (at the end of the experiment, DPI 7) and counts of different immune cells. **b** Correlation between the relative abundance of bronchoalveolar lavage fluid bacterial genera (7 days post-influenza virus infection) and counts of immune cells and titers of IgA and IgG. Only significant correlation was shown, with the direction and strength of correlation (correlation efficient) shown by the heatmap scale

The abundance of some genera of colonic bacteria (7 days post-influenza virus infection) and fecal microbiota (7 days post-influenza virus infection) correlated with the frequency/population of different immune cells (Fig. 4a). In the colonic microbiota, *Enterococcus* negatively correlated with T-helper/memory cell population, while *Citrobacter* and *Turicibacter* negatively correlated with myeloid cell population. Non-T-cell frequency correlated with different genera, including positive correlation with *Morganella* but negative correlation with *Pseudoramibacter, Shigella,* and one unclassified genus of *Clostridiales*. More genera of the fecal microbiota correlated with immune cells: Myeloid cell counts positively correlated with *Streptococcus, Parabacteroides*, *Enterococcus*, *Odoribacter*, *Eubacterium*, *Butyricimonas*, and one unclassified genus of *Morgibacteriaceae*, but negatively correlated with *Citrobacter* and *Turicibacter*. Twelve genera of fecal bacteria had correlations with non-T-cell population, including a positive correlation with *Streptococcus, Parabacteroides*, and *Enterococcus*, and a negative correlation with genera such as *Sutterella* and *Ruminococcus*,  and six other genera each of RF32 and *Barnesiellaceae*. Cytotoxic T-cell frequency also appeared correlated with some genera of fecal bacteria, including a negative correlation with *Streptococcus, Parabacteroides*, *Enterococcus*, and *Corynebacterium*, and a positive correlation with *Shigella* and *Citrobacter*. Furthermore, T-helper/memory cell population positively correlated with fecal *Peptococcus*, *Proteus*, *Robinsoniella*, and one unclassified genus of *Peptostreptococcaceae* and negatively correlated with fecal *Bifidobacterium*, *Slackia*, *Blautia*, *Collinsella*, *Allobaculum*, and one unclassified genus each in the families of *Christensenellaceae* and *Barnesiellaceae*. The relative abundance of some bacterial genera in bronchoalveolar lavage fluid correlated with the BAL fluid IgG and IgA titers, BAL fluid immune cell counts, TBLN MINCs immune cell counts, and myeloid cell counts (Fig. 4b).

Specifically, the relative abundance of *Chryseobacterium* positively correlated with the titers of both BAL fluid IgG and IgA titers, while that of *Moreaxella* negatively correlated with the titer of only BAL fluid IgG. Non-T-cell (CD3-) population in BAL fluid positively correlated with *Anaerotruncus* and one unclassified genus of *Sinobacteraceae*, while the BAL fluid myeloid cell population positively correlated with *Pseudomonas*, *Bosea*, *Sediminibacterium*, *Mesorhizobium*, and one unclassified genus of *Bradyrhizobiaceae*, but negatively correlated with *Bifidobacterium*, *Lactobacillus*, *Ruminococcus*, one unclassified genus each of the class *Clostridiales* and the candidate family RF32. With respect to TBLN MNCs immune cells, T-helper/memory cell population negatively correlated with *Methylobacterium*, *Bergeyella*, and one unclassified genus of *Phyllobacteriaceae*, while the cytotoxic T-cell frequency had a positive correlation with *Blautia* and *Oscillospira* but a negative correlation with *Burkholderia*, *Faecalibacterium*, *Proteus*, *Peptoniphilus*, and *Klebsiella*. In the TBLN MNCs, we found myeloid cells, non-T cells (CD3-) positively correlated with *Anaerotruncus*, while myeloid cells negatively correlated with *Eggerthella*, *Staphylococcus*, and *Bergeyella*.

**Fig. 5:**
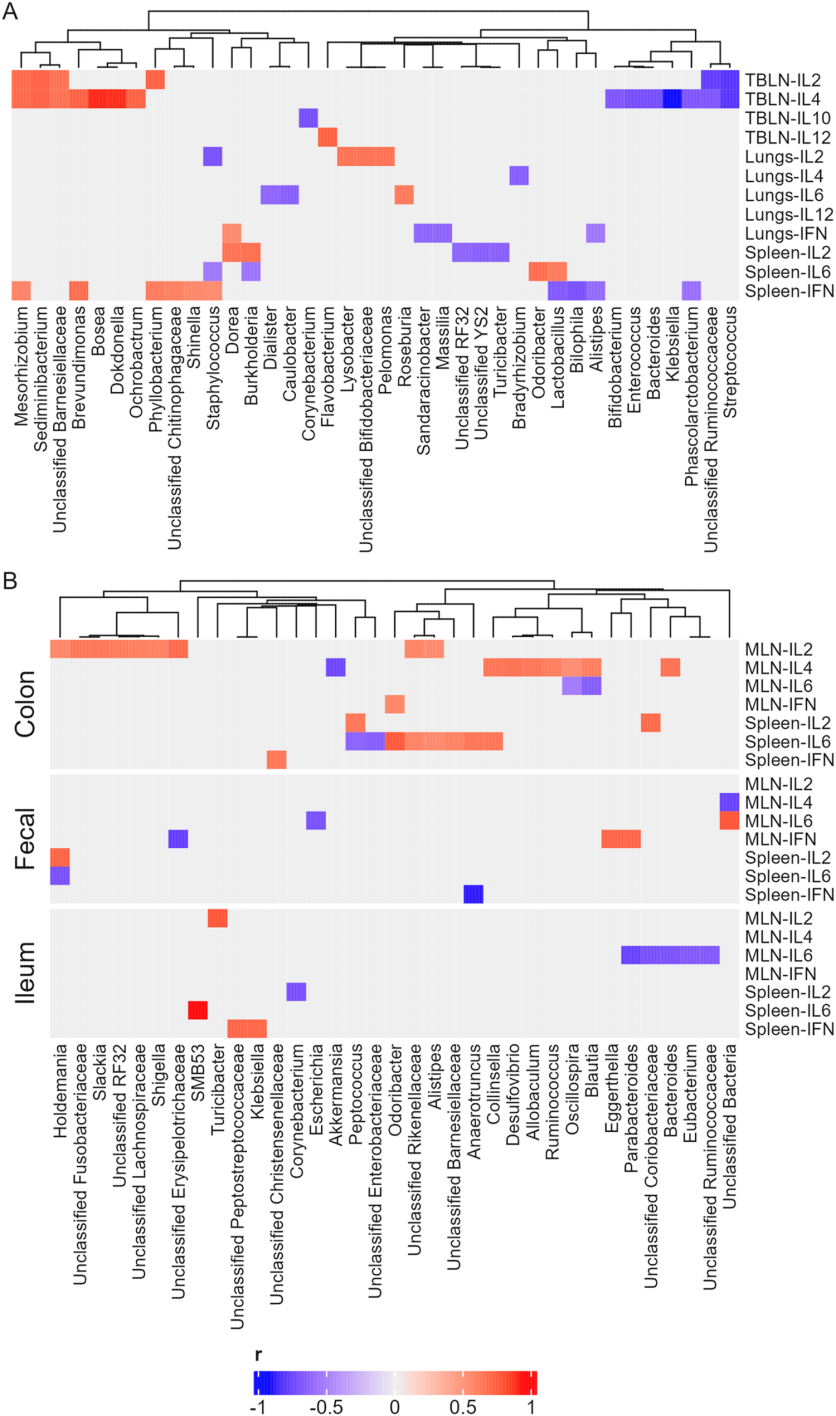
Kendall’s rank correlation between the relative abundance of bacterial genera of the piglet microbiota and expression of cytokines. **a** Correlation between the relative abundance of bronchoalveolar lavage fluid bacterial genera (at the end of the experiment, 7 days post-influenza virus infection) and expression of cytokines. **b** Correlation between the abundance of colonic, fecal, and ileal bacterial genera (at the end of the experiment, DPI 7) and expression of cytokines. Only significant correlation was shown, with the direction and strength of correlation (correlation efficient) shown by the heatmap scale

In the TBLN, IL-2 expression correlated with the most genera of bronchoalveolar lavage fluid bacteria, including positive correlation with *Mesorhizobium*, *Sediminibacterium*, *Brevundimonas*, *Bosea*, *Dokdonella*, and *Ochrobactrum* and negative correlation with *Bifidobacterium, Enterococcus*, *Bacteroides*, *Klebsiella*, *Phascolarctobacterium*, and *Streptococcus* (Fig. 5a). The spleen IFNγ correlated with the second most genera of bronchoalveolar lavage fluid bacteria, including a positive correlation with *Mesorhizobium, Brevundimonas, Phyllobacterium, Shigella,* and *Staphylococcus* and a negative correlation with *Lactobacillus, Bilophila, Alistipes,* and *Phascolarctobacterium*. Interestingly, several genera of bronchoalveolar lavage fluid were correlated with the expression of two or more different cytokines, including *Mesorhizobium*, *Sediminibacterium*, *Brevundimonas, Phyllobacterium, Staphylococcus, Dorea*. *Burkholderia*, *Lactobacillus*, *Alistipes*, and *Streptococcus*.

A total of 22 genera and 13 unclassified or candidate genera of fecal and intestinal (ileal and colonic) bacteria correlated positively and/or negatively with the expression of one or more cytokines (Fig. 5b). More genera of the colonic bacteria had a correlation, mostly positive correlation, with the cytokine expression than the ileal or fecal bacteria. Notably, in the MLN, IL-2 expression correlated positively with *Holdemania*, *Slackia*, *Shigella*, and *Alistipes* and several unclassified genera, while in MLN IL-4, the expression was positively correlated with *Collinsella*, *Desulfovibrio*, *Allobaculum*, *Ruminococcus*, *Oscillospira*, *Blautia*, and *Bacteroides*. The expression of IL-6 in the spleen also correlated with numerous genera of bacteria, including a negative correlation with *Peptococcus*, and a positive correlation with *Odoribacter*, *Alistipes*, *Anaerotruncus*, *Collinesella*, and three unclassified genera.

## Discussion

Changes in intestinal bacterial structure can influence disease outcomes in the respiratory tract away from the gut. It has been suggested that gut microbiota can modulate the antiviral immune responses, potentially mediated by metabolites like short-chain fatty acids [43]. In addition, mononuclear phagocytes in the intestine and respiratory tract can sample antigens in the lumen and activate adaptive immunity to promote the clearance of viruses and other pathogens [43]. Our data suggest that infant microbiota likely influences the influenza virus load in the respiratory tract, mediated by differentially modulated innate and Th1 immune-inducing factors but not Th2 factors, because antibody responses in IFM humanized piglets were not altered.

The CD172a is expressed on all the pig monocytes, granulocytes, and dendritic cells but not on T and B cells [44, 45]. The commensal microbiota composition critically regulates the generation of virus-specific CD4 and CD8 T cells and antibody responses following influenza virus infection [11].

The interplay between the gastrointestinal microbiota, invasive viruses, and host immunology is complex and not fully characterized; however, recent evidence shows that the microbiota plays an important role in the pathogenesis of viral diseases [46]. The gut microbiota provides necessary signals for the expression of cytokines, such as pro-IL-1β and pro-IL-18, and following influenza virus infection, the inflammasome activation leads to homing of immune cells from the lung to the draining lymph nodes for activating cell-mediated immune responses [11]. In this study, we observed vast differences in the expression of mRNA of cytokines IL-6, IL-2, and IL-4 in the TBLN and MLN of piglets colonized with RIFM and UIFM, suggesting the likely role played by the diverse microbial groups. In one study, some members of nose/throat microbiota were found to be associated with susceptibility to influenza virus infection, but it is transitory among young children and stable among adults, suggesting the upper respiratory tract microbiota may be a potential target for reducing the burden of influenza viruses [47]. Significant correlations were observed between cytokine gene expression levels and relative abundances of several bacteria in trachea of influenza-infected turkeys. For example, IFNγ/λ and IL-6 gene expression levels were correlated positively with *Staphylococcus* and *Pseudomonas* abundance, and negatively correlated with abundance of *Lactobacillus* [48].

A systematic review revealed the positive role played by probiotics on immunity to influenza vaccines in human clinical trials through significantly improving hemagglutination inhibition antibody titers [49, 50]. The gut microbiota signals to the lung stromal cells keep the latter in an IFN-primed state, resulting in protection from influenza virus infection [51]. A probiotic strain of *L. paracasei* reduces the susceptibility to influenza infection by reducing inflammatory cell infiltrates in the lungs and promoting viral clearance [52]. In the lungs and MLN of piglets, we found modulation in the IFNγ gene expression influenced more by UIFM than RIFM. Overall, the expression of cytokines and pro-and anti-inflammatory responses to acute respiratory viral infection is potentially influenced by colonized diverse infant gut microbiota.

Immune cells and molecules present in mucosal tissues of the respiratory and intestinal tracts are connected throughout the body [53]. Thus, bilateral interactions of intestinal microbiota and its metabolites should influence, as well as be influenced by, the course of influenza virus infection [43]. The H7N9 influenza virus infection in people reduces intestinal microbiota diversity and increases colonization by *E. coli* and *Enterococcus faecium* [54]. Type I IFNs circulate via the bloodstream from the respiratory tract to the gut during influenza infection, and high levels of type I IFNs trigger IL-17 production leading to the production of several proinflammatory cytokines and chemokines [43]. These sequential events may allow opportunistic microorganisms to gain a foothold and even thrive in mucosal tissues [43].

The relative abundance of the detected bacterial phyla did not change in response to the viral infection two days post-infection, but at 4 and 7 days post-viral infection, Proteobacteria nearly diminished in both piglet groups. This contradicts a previous study that reported increased Proteobacteria in response to influenza virus infection in an influenza mouse model [55]. Mouse and humans differ in their gut microbiota [56], and we humanized the Gn piglet gut with infant fecal microbiota. It is plausible that gut microbiota can respond to influenza virus infection in a gut microbiota-dependent manner. Interestingly, Bacteroidetes filled the void left by Proteobacteria in the rural group, whereas Firmicutes occupied the niche left by Proteobacteria in the urban group. Although this study lacks a control group that was not infected with the influenza virus, the significant shifts at the phylum level within several days of the viral infection suggest that influenza virus infection can affect fecal microbiota. Further, the differences in phylum-level shifts between the two piglet groups also suggest that the influenza virus infection can impact the gut microbiota in a gut microbiota-dependent manner. This axiom, however, needs to be verified with phylum-specific qPCR.

Very little is known about how gut microbiota responds to influenza virus infection, but the observed increase in *Klebsiella*, whose members have polysaccharide-based capsules and colonize the nasal, oral, and intestinal tracts, concurs with a recent study that reported *Klebsiella* increase following influenza virus infection in piglets whose gut microbiota was humanized with fecal microbiota from healthy or obese donors [57]. *Clostridium*, *Klebsiella*, and *Streptococcus* all contain infectious species. Influenza virus infection increased *Salmonella* colonization in the gut of mice [55]. We thus interpret the increase of these genera to potentially represent an increased risk of gut infection by species of these genera, especially *Clostridium*, upon influenza infection.

A growing number of studies indicated that the microbiota colonizing the upper respiratory tract consisting of a high abundance of *Streptococcus*, *Neisseria,* and *Haemophilus* and a low abundance of *Moraxella*, *Staphylococcus*, *Corynebacterium*, and *Dolosigranulum* in healthy children following influenza A virus infection [3]. In another study, *Streptococcus* was significantly decreased in the nasopharynx following influenza virus infection, and decision tree analysis indicated that *Ralstonia* and *Acidobacteria* could discriminate microbial samples in healthy and influenza-infected animals with high accuracy [58].

Although some of the genera are commensal bacteria and correlation does not imply any causality, the correlations between the genera that contain infectious species or strains and expression of cytokines and immune cell counts are of potential clinical interest. These include the correlation between cytokine gene expression with *Dosea, Dokdonella, Ochrobactrum, Enterococcus, Klebsiella, Streptococcus, Shigella,* and *Staphylococcus* of bronchoalveolar lavage fluid. Also interestingly, some genera were correlated with both cytokine gene expression, immune cell counts, and immunoglobins abundance. Notably for bronchoalveolar lavage fluid microbiota, the relative abundance of the genera *Sediminibacterium*, *Bosea*, and *Mesorhizobium* was positively correlated with both TBLN IL-4 expression and BAL cells myeloid cell counts. On the contrary, the relative abundance of *Bifidobacterium* was negatively correlated with TBLN IL-4 expression and BAL cells myeloid cell counts. In addition, the relative abundance of the genus *Klebsiella* was negatively correlated with both TBLN IL-4 expression and TBLN cytotoxic T cells population. The genus of *Alistipes* was negatively correlated with both spleen and lung IFNγ production. Similarly, for the fecal microbiota, the genus of *Parabacteroides* was positively correlated with both the MLN IFNγ expression and the abundance of myeloid cells and non-T cells. However, for the colon microbiota, none of these genera was found to correlate with both cytokine expression and immune cells or immunoglobins abundance. Future research is needed to determine whether these bacterial genera play any significant role in regulating the immune response to influenza virus infection. Further studies should also focus on revealing the long-term impact of colonization of human microbes in a Gn piglet model on their stability, structure, and metabolites after influenza virus infection.

## Data Availability

The datasets used and/or analyzed during the current study are available from the corresponding author on reasonable request. The sequences reported in this paper have been deposited in NCBI with accession number: PRJNA1012342.
